# Use of a combination of diaphragmatic ultrasound and muscle relaxation monitoring in predicting post-extubation adverse respiratory events among elderly patients in an anesthesia intensive care unit

**DOI:** 10.1186/s12890-023-02791-z

**Published:** 2023-12-12

**Authors:** Lun Huang, Bo Xia, Lei Cheng, Xian-Wen Hu, Li-Dong Zheng, Feng Cheng

**Affiliations:** 1 Graduate Department, Bengbu Medical College, Bengbu, 233030 China; 2 Department of Anesthesiology, Lu’an Hospital of Anhui Medical University, No. 21 of Wanxi West Road, Lu’an, 237005 China; 3 Department of Anesthesiology, The Second Hospital of Anhui Medical University, Hefei, 230601 China

**Keywords:** Diaphragm ultrasound, Muscle relaxation monitoring, Old age, Respiratory adverse events

## Abstract

**Objective:**

The purpose of this study was to examine the feasibility of using a combination of diaphragmatic ultrasound and muscle relaxation monitoring in predicting adverse respiratory events after extubation among elderly patients in an anesthetic intensive care unit (AICU).

**Methods:**

The study participants were 120 elderly patients who were in the AICU after laparoscopic radical resection for colorectal cancer. Based on whether there were critical respiratory events (CREs) after extubation, they were divided into the adverse event group and the non-adverse event group. We used logistic regression to identify factors influencing the occurrence of CREs post-extubation in elderly patients. Using the receiver operating characteristic (ROC) curve, we analyzed the value of each indicator in predicting CREs post-extubation.

**Results:**

We included 109 patients in the final analysis. In the adverse event group (n = 19), the age, proportion of females, and proportion of preoperative respiratory diseases were higher than in the non-adverse event group (n = 90). The muscle relaxation value, quiet breathing diaphragmatic excursion during extubation (DE-QB), deep breathing diaphragmatic excursion during extubation (DE-DB), and deep breathing diaphragmatic thickening fraction during extubation (DTF-DB) of patients in the adverse event group were significantly lower than those in the non-adverse event group (P < 0.05). Using binary logistic regression analysis, we identified muscle relaxation value, DE-DB, and DTF-DB during extubation as significant predictors of CREs post-extubation in elderly patients (P < 0.05). The area under the curve (AUC) of the combination of the muscle relaxation value, DE-DB, and DTF-DB during extubation for predicting CREs after extubation in elderly patients was 0.949, which was higher than that of any single indicator.

**Conclusion:**

The combination of diaphragmatic ultrasound and muscle relaxation monitoring was more accurate in predicting CREs post-extubation among elderly patients in the AICU.

## Introduction

In recent years, there has been a rise in the number of surgeries done on elderly patients. Such procedures for most elderly patients are often complicated due to complex internal and surgical diseases prior to the operation [1] and their frail state. Their cardiopulmonary function reserve capacity is generally low, and most of them are shifted to the anesthesia intensive care unit (AICU) post-surgery. The AICU is a ward that is essential for the monitoring of critically ill patients after anesthesia and post-surgery and ensures perioperative safety and rapid recovery of critically ill patients after the surgery, as well as reduction of postoperative complications. [2].

Extubation after surgery is a risky event during the period of recovery from general anesthesia. [3] Improper extubation timing can easily cause critical respiratory events (CREs). Elderly patients, in particular, have an increased risk of CREs after extubation. CREs can have an adverse impact on the physiology and psychology of elderly patients and seriously affect their prognosis. Timely, accurate, and effective extubation can reduce the incidence of postoperative respiratory adverse events while also reducing the use of anesthetic drugs, increasing the comfort and safety of elderly patients in the AICU, facilitating rapid rehabilitation, and reducing pain.

At present, successful extubation post-surgery mainly depends on the recovery of muscle relaxation. The train of four ratio (TOFr) is a commonly used measure of residual muscle relaxation. [4] Muscle relaxation monitoring with train of four (TOF) stimulation is mainly aimed at peripheral small muscle groups, [5] and its muscle strength recovery is not exactly identical to the muscle group that provides for the human respiratory dynamic. [6].

Diaphragmatic ultrasound is a procedure that has been continuously refined since it was first used in clinical practice. Diaphragm dysfunction, which accounts for 60–70% of human respiratory dynamics, [7] is closely related to postoperative complications. The recovery of the diaphragm is particularly important when conducting extubation. Diaphragm recovery and muscle relaxation monitoring will affect the respiration of elderly patients after extubation, so we think that diaphragm ultrasound combined with muscle relaxation monitoring can more effectively predict the occurrence of adverse respiratory events post-extubation in elderly patients.

In this study, we explored the utility of a combination of diaphragmatic ultrasound and muscle relaxation monitoring in predicting the extubation outcome of elderly patients in the anesthesia intensive care unit following laparoscopic radical resection for colorectal cancer. We also sought to identify more accurate predictors in order to reduce adverse respiratory events post-extubation and ensure the post-operative safety of elderly patients.

## Materials and methods

### Study participants

We included a total of 120 patients who were admitted to the anesthesia intensive care unit of the Liu’an People’s Hospital, China, following laparoscopic radical resection for colorectal cancer between February 2022 and March 2023. According to the occurrence of adverse respiratory events post-extubation, they were divided into the adverse event group and the non-adverse event group.

Inclusion criteria: (1) Patients with American Society of Anesthesiologists (ASA) grade II–III health status; (2) Age ≥ 65 years. Exclusion criteria: (1) History of previous neuromuscular disease or history of diaphragmatic dysfunction; (2) New York Heart Association (NYHA) > III, Chronic Kidney Disease (CKD) > IV, Child-Pugh C; (3) Known or suspected allergy to drugs used during anesthesia; (4) Inability to perform muscle relaxation monitoring; (5) Unstable hemodynamic status.

This study was approved by the Ethics Committee of the Liu’an People’s Hospital with the approval number: [2022LL (research) 001], and informed consent was obtained from all the family members of respondents in the study.

### Research methods

This study is a cohort study. According to the occurrence of respiratory adverse events after extubation, patients are divided into adverse events group and no adverse events group. The same anesthesiologist measured the diaphragm parameters of the standard patients before operation. After entering the operating room, the patients were monitored for muscle relaxation and sent to AICU after the operation. After judging the condition of the patients, extubation was performed by AICU extubation doctor. The muscle relaxation values were recorded by independent personnel, and the diaphragm parameters were measured by the same anesthesiologist at the same time. In this study, neither the AICU extubation doctor nor the anesthesiologist who measured the diaphragm knew the muscle relaxation value at the time of extubation. We used PASS v21.0.3 to calculate sample size, the significance level α value is 0.05 on both sides, and the sample drop rate of 10% is considered. The required sample size is 118 cases.

#### Preoperative visit, anesthesia induction, and maintenance

Before the surgery, medical staff visited the patients who were to be shifted to the AICU, and the patients were taught how to do respiratory functional exercises. After entering the operating room, the patient’s diaphragmatic muscle value was measured, peripheral veins were opened, ECG monitoring, arterial and internal jugular vein puncture, and pressure measurement, as well as bispectral index score (BIS) monitoring, were performed. Etomidate (0.15–0.3 mg/kg) and sufentanil (0.1–2 ug/kg) were used for anesthesia induction. When the patient’s BIS was < 60, the muscle relaxation monitors were connected and set to zero. Cisatracurium 0.2 mg/kg was administered. Tracheal intubation was performed when the TOFr count was 0. Propofol (4–12 mg/kg/h) and remifentanil (0.25–2 ug/kg/min) were maintained during the surgery. The infusion rate of cisatracurium was adjusted according to the muscle relaxation value perioperatively, and post-surgery, the patients were shifted to the AICU.

#### Muscle relaxation monitoring

In the operating room, the patient’s forearm was abducted, and the skin was cleaned with alcohol wipes. The red and black electrodes of the muscle relaxation monitor (Veryark-TOF, Guangxi Veryark Technology Co., Ltd.) were fixed on the electrode sheet and attached to both sides of the ulnar nerve at a distance of about 2 cm apart. At the same time, the muscle tension sensor was placed on the purlicue, and the temperature sensor was fastened around the thumb medially. Calibration was performed after the anesthesia took effect, and cisatracurium was administered after the calibration. The TOFr mode was used during the surgery, and monitoring was done at five-minute intervals. The arm was kept warm, and after the patient was shifted to the AICU, the monitoring time interval was adjusted to 30 s. In the AICU, the value of muscle relaxation immediately after extubation was recorded by an independent person.

#### Diaphragmatic ultrasound

In the operating room, the diaphragmatic value was measured before induction of anesthesia and immediately after extubation. The same person measured the diaphragmatic value of the patient using an ultrasound machine (TE7Pro, Shenzhen Mindray Biomedical Electronics Co., Ltd.). With the patient in the supine position, the low-frequency probe was placed at the junction of the middle line of the right clavicle and the lower edge of the costal arch to scan the arc-shaped hyperechoic tissue, that is, the diaphragm muscle, and was switched to M-mode ultrasound. The patient was asked to measure the vertical distance between the peak and the trough during quiet breathing and deep breathing, that is, diaphragmatic excursion-quiet breathing (DE-QB) and diaphragmatic excursion-deep breathing (DE-DB), respectively. The high-frequency probe was placed at the intersection of the right anterior axillary or midaxillary line and the lower edge of the 8–10 ribs. The patient was encouraged to breathe calmly and deeply. The thickness of the diaphragm at the end of inspiration and the end of expiration in the same respiratory cycle was measured as follows:

Diaphragmatic thickening fraction = (end-inspiratory diaphragm thickness - end-expiratory diaphragm thickness)/end-expiratory diaphragm thickness.

We also measured diaphragmatic thickness fraction-quiet breathing (DTF-QB) and diaphragmatic thickness fraction-deep breathing (DTF-DB).

#### AICU treatment and extubation

The patients who were shifted to the AICU were continuously monitored for muscle relaxation. It is considered that extubation in the operating room of elderly patients after laparoscopic surgery may cause adverse events and affect the prognosis of the patients. The AICU anesthesiologist involved in this study evaluated the patient’s breathing, level of consciousness, and muscle strength recovery conditions, for example, the respiratory rate is less than 30 beats / min, clear consciousness can be instructed to open eyes, swallow and cough reflex recovery, look up for more than 5 s, a strong handshake and other evidence. Immediately after extubation, the Observer Assessment of Alertness/Sedation Scale (OAA/S) score was recorded. [8] Independent personnel recorded the value of muscle relaxation during extubation. Immediately after extubation, the diaphragmatic value of the patient was measured again by the same anesthesiologist who performed the diaphragmatic ultrasound on the patient before surgery. Patients were divided into the adverse event group and the non-adverse event group based on the presence or absence of CREs at 30 min after extubation (diagnostic criteria are shown in Table 1). [9].

**Table 1:** Diagnostic criteria for adverse respiratory events

Adverse respiratory events
Upper respiratory tract obstruction requires manual intervention
Mild to moderate hypoxemia (blood oxygen saturation of 90–93%)
Severe hypoxemia (blood oxygen saturation < 90%)
Signs of respiratory distress or respiratory failure (respiratory rate > 20 times /min, with the involvement of accessory respiratory muscle).
Unable to follow instruction to perform deep breathing
Symptoms of upper respiratory muscle weakness (difficulty breathing, swallowing, speaking)
Repeated intubation
Reflux and aspiration after extubation (gastric solute was found in oropharynx)

#### Monitoring indicators

##### Main observation indicators

Muscle relaxation value during extubation, diaphragmatic value during extubation, and adverse respiratory events 30 min after extubation.

##### Secondary observation indicators

Age, gender, proportion of preoperative respiratory diseases, ASA grade, BMI, pneumoperitoneum duration, duration of anesthesia, cisatracurium dosage, liquid intake and output, heart rate during extubation, mean arterial pressure during extubation, preoperative diaphragmatic ultrasound parameters, extubation pH, partial pressure of carbon dioxide (PCO_2_), partial pressure of oxygen (PO_2_), sedation score, pain score.

### Statistical analysis

We used PASS v21.0.3 to calculate sample size, the significance level α value is 0.05 on both sides, and the sample drop rate of 10% is considered. We used SPSS 27.0 statistical software for data analysis. Measurement data with a normal distribution were expressed as x¯±s, and the t-test was used for comparison between groups. Enumeration data were expressed as frequency or percentage, and the chi-square test was used for comparison. Regression analysis was performed on statistically significant indicators to screen out the risk factors for CREs post-extubation. The receiver operating characteristic (ROC) curve was drawn for each risk factor to predict its ability to evaluate CREs after extubation. A *P* value of < 0.05 was considered statistically significant.

## Results

### Comparison of the sociodemographic profiles of the two groups of elderly patients

There were 109 patients in the final analysis (we excluded 8 cases of laparotomy and 3 cases of rejection). The details of the general clinical information are shown in Table 2. The incidence of adverse respiratory events after extubation was 17.4%; the proportion of patients with a muscle relaxation value < 0.9 during extubation was 45.9%; and the proportion of patients with a muscle relaxation value < 0.6 during extubation was 4.6%. The proportion of females, older patients, and those with preoperative respiratory diseases in the adverse event group were higher than those in the non-adverse event group. Patients in the adverse event group had significantly lower muscle relaxation value during extubation, quiet breathing diaphragmatic excursion during extubation, deep breathing diaphragmatic excursion during extubation, and deep breathing diaphragmatic thickening fraction during extubation than those in the non-adverse event group (P < 0.05). (Table 2).

**Table 2:** Comparison of sociodemographic profiles of the two groups of elderly patients in the AICU

Variable	Total (n = 109)	Adverse events		χ^2^/t	P value
		No (N = 90)	Yes (N = 19)		
Age (in years)	76.27 ± 5.28	75.72 ± 5.17	78.84 ± 5.12	2.39	0.018
Gender(Male/Female)	59/50	53/37	6/13	4.71	0.030
Proportion of preoperative respiratory diseases (%)	20(18.3%)	12(13.3%)	8(42.1%)	6.85	0.009
ASA grade (II–III)	60/49	50/40	10/9	0.054	0.816
BMI (kg/m^2^)	21.99 ± 3.24	22.13 ± 3.36	21.32 ± 2.55	0.990	0.324
Pneumoperitoneum duration (min)	211.07 ± 61.27	212.96 ± 59.97	202.11 ± 68.11	0.700	0.485
Duration of anesthesia (min)	272.81 ± 63.74	272.82 ± 62.20	272.74 ± 72.44	0.005	0.996
Cisatracurium dosage (mg)	35.88 ± 8.17	35.96 ± 8.18	35.52 ± 8.29	0.215	0.830
Liquid intake and output (ml)	1376.70 ± 449.07	1383.11 ± 413.98	1346.31 ± 601.19	0.323	0.747
Muscle relaxation value during extubation (%)	83.97 ± 11.36	86.40 ± 9.69	72.47 ± 11.89	5.465	<0.001
Heart rate during extubation (times/min)	72.66 ± 5.97	72.44 ± 5.68	73.68 ± 7.28	0.822	0.413
Mean arterial pressure during extubation (mmHg)	83.70 ± 7.06	83.54 ± 7.50	84.47 ± 4.51	0.519	0.605
pH during extubation	7.36 ± 0.03	7.36 ± 0.03	7.37 ± 0.04	0.776	0.440
PO_2_ during extubation (mmHg)	256.38 ± 85.96	252.21 ± 83.13	276.11 ± 98.32	1.102	0.273
PCO_2_ during extubation (mmHg)	41.42 ± 4.32	41.63 ± 4.05	40.42 ± 5.44	1.11	0.268
Observer Assessment of Alertness/Sedation score (OAA/S)	2.44 ± 0.50	2.53 ± 0.51	2.42 ± 0.50	0.826	0.411
Pain score (VAS score)	1.91 ± 1.00	1.91 ± 1.02	1.93 ± 0.96	0.084	0.933
Preoperative quiet breathing diaphragmatic excursion (cm)	2.17 ± 0.30	2.16 ± 0.30	2.22 ± 0.33	0.789	0.432
Preoperative deep breathing diaphragmatic excursion (cm)	3.96 ± 0.61	3.95 ± 0.64	4.04 ± 0.44	0.592	0.555
Quiet breathing diaphragmatic excursion during extubation (cm)	1.84 ± 0.15	1.86 ± 0.15	1.76 ± 0.13	2.723	0.008
Deep breathing diaphragmatic excursion during extubation (cm)	3.13 ± 0.49	3.23 ± 0.47	2.65 ± 0.28	5.254	<0.001
Preoperative quiet breathing diaphragmatic thickening fraction (%)	33.32 ± 7.85	33.93 ± 8.07	30.42 ± 6.04	1.791	0.076
Preoperative deep breathing diaphragmatic thickening fraction (%)	83.25 ± 13.77	83.08 ± 14.63	84.05 ± 8.80	0.279	0.781
Quiet breathing diaphragmatic thickening fraction during extubation (%)	27.90 ± 10.62	28.34 ± 11.01	25.79 ± 8.45	0.953	0.343
Deep breathing diaphragmatic thickening fraction during extubation (%)	66.62 ± 13.79	69.17 ± 13.31	54.58 ± 8.90	4.557	<0.001

### Logistic regression analysis of factors influencing CREs post-extubation

We considered the occurrence of CREs after extubation (no adverse event = 0, adverse event = 1) as the dependent variable and conducted a logistic regression analysis with age, gender (male = 0, female = 1), preoperative respiratory disease(no = 0, yes = 1), TOFr, DE-QB, DE-DB, and DTF-DB during extubation as covariates. The prediction model equation is Logit (P) = 22.634–3.890×deep breathing diaphragmatic excursion during extubation-0.102×deep breathing diaphragmatic thickening fraction during extubation-0.117×muscle relaxation value during extubation. The results showed that TOFr, DE-DB, and DTF-DB during extubation were independent risk factors that significantly influenced CREs after laparoscopic extubation in the elderly (P < 0.05). (Table 3)

**Table 3:** Logistic regression analysis of factors influencing CREs post-extubation

Factor	β	S.E	Wald	P值	OR	95%CI	
						Lower limit	upper limit
Age	0.124	0.086	2.067	0.151	1.132	0.956	1.341
Preoperative complicated respiratory diseases	1.029	0.918	1.257	0.262	2.798	0.463	16.904
Gender	-0.802	0.882	0.827	0.363	0.448	0.080	2.527
Quiet breathing diaphragmatic excursion during extubation	-3.920	3.820	1.053	0.305	0.020	0.001	35.388
Deep breathing diaphragmatic excursion during extubation	-3.890	1.729	5.060	0.024	0.020	0.001	0.606
Deep breathing diaphragmatic thickening fraction during extubation	-0.102	0.046	4.850	0.028	0.903	0.825	0.989
Muscle relaxation value during extubation	-0.117	0.041	8.266	0.004	0.890	0.822	0.963
Constant	22.634	12.649	3.202	0.074	/	/	/

### ROC curve analysis of the combination of diaphragmatic parameters and muscle relaxation values to predict successful ventilator withdrawal in patients

**Fig. 1:**
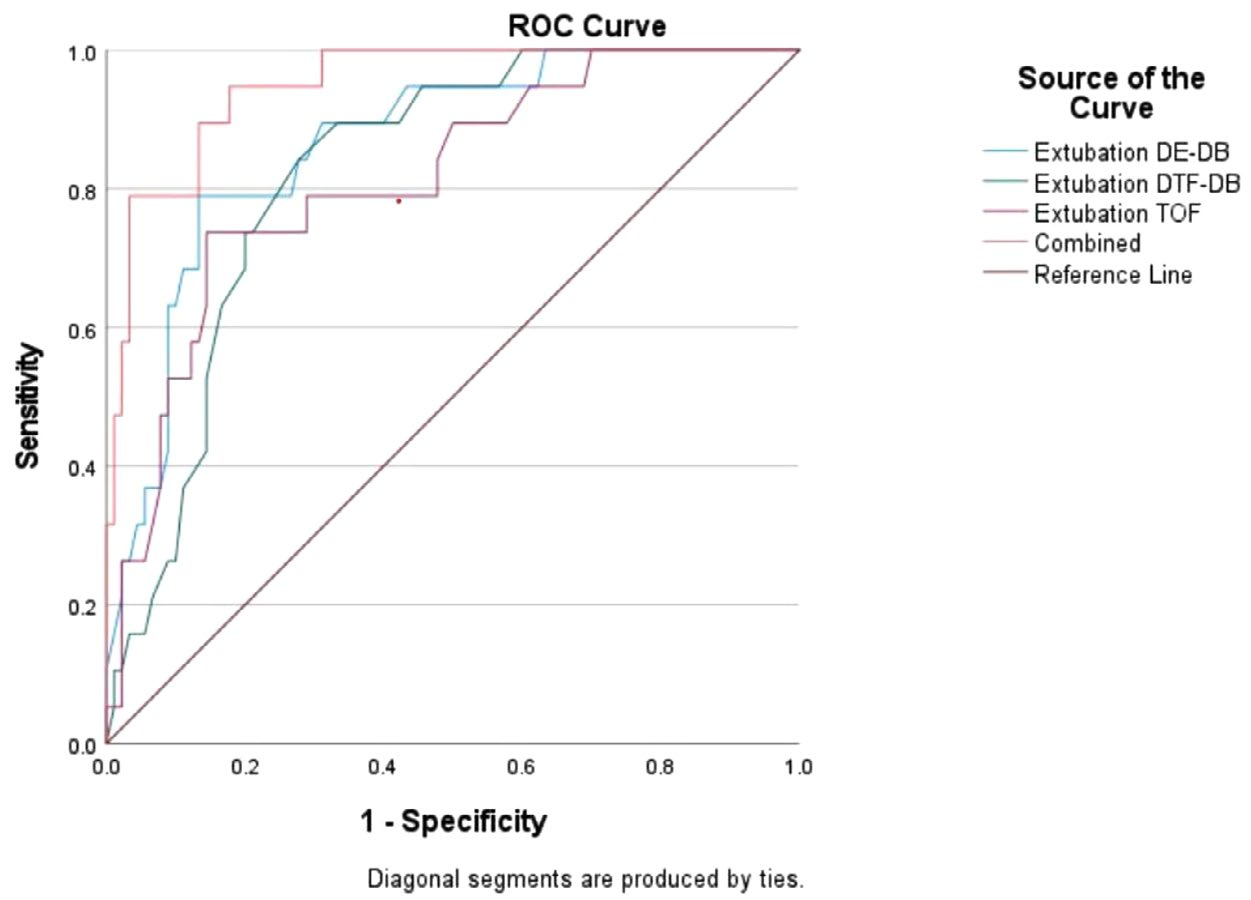
ROC curve of muscle relaxation value and diaphragmatic parameters alone and in combination to predict CREs post-extubation in elderly patients

The three indicators, namely, muscle relaxation value, DE-DB, and DTF-DB during extubation, were useful in predicting CREs post-extubation in elderly patients undergoing laparoscopy, and they could be used in combination to predict CREs after extubation in these patients. The cut off of muscle relaxation monitoring is 74.15. The cut off of DE-DB is 2.83 cm.The cut off of DTF-DB is 62.5%.The ROC curves of the three indicators alone and in combination to predict adverse events are displayed in Table 4; Fig. 1.

**Table 4:** ROC curve analysis of each indicator to predict adverse events post-extubation in elderly patients undergoing laparoscopy

Indicator	AUC	95%CI	The best diagnostic point	Sensitivity %	Specificity %	Accuracy %	P
Deep breathing diaphragmatic excursion during extubation	0.865	0.782 ~ 0.948	2.83 cm	78.9	86.7	65.6	<0.001
Deep breathing diaphragmatic thickening fraction during extubation	0.825	0.74 ~ 0.91	62.5%	84.2	72.2	56.4	<0.001
Muscle relaxation value during extubation	0.813	0.708 ~ 0.917	74.15	73.7	85.6	59.3	<0.001
Combined indicators	0.949	0.906 ~ 0.993	0.110	94.7	82.2	76.9	<0.001

## Discussion

Patients in the AICU usually have complications due to other systemic diseases, poor intraoperative conditions, older age, and postoperative adverse events. [2] Additionally, extubation after general anesthesia is a high-risk perioperative event. If the extubation is done too early, it can result in respiratory adverse events, while late extubation can increase the risk of complications such as pulmonary infection. Accurate and timely extubation during resuscitation can lessen the anesthetic intake and prevent ventilator-related injury. Therefore, this procedure is a critical link in the treatment of patients in the AICU, especially with the growing popularity of accelerated rehabilitation post-surgery. Accurately predicting adverse respiratory events after extubation in elderly patients in the AICU and early interventions can significantly benefit patients by reducing pain, improving their comfort, and minimizing postoperative complications.

Clinicians who perform extubation generally rely on their clinical experience; that is, they perform the extubation based on the recovery of muscle strength by judging the patient’s grip strength, tongue extension, leg lifting, and other actions. Physiological changes in the elderly [10] cause the metabolism of muscle relaxation drugs in elderly patients to differ from that in young people, and this makes them more vulnerable to postoperative residual curarization (PORC). Residual muscle relaxation can induce serious harm in elderly patients and can increase the incidence of postoperative pulmonary complications, [11] which is not favorable for the prognosis in such patients. Currently, extubation is considered safe when muscle relaxation monitoring TOFr is > 0.9. [12].

In this study, the clinician was blinded to the patient’s muscle relaxation value when performing the extubation. We found that about 45.9% of the patients had a TOFr < 0.9 when undergoing extubation, and this was slightly lower than the residual rate of muscle relaxation in the study by Murphy et al. [10] This confirms that there are obvious deficiencies in extubation performed based on experience. We found that 4.6% of the patients had a TOFr < 0.6, which was significantly lower than the 22.9% incidence of TOFr < 0.6 after abdominal surgery reported by Yu et al. [13] This difference may be due to the safety monitoring provided in the AICU, due to which, after a period of sedation, the patients shifted to the AICU had a more complete recovery of muscle relaxation, allowing for a more accurate timing of extubation. Furthermore, the ROC curve analysis confirmed that extubation under muscle relaxation monitoring could predict the occurrence of respiratory adverse events post-extubation in elderly patients after laparoscopic radical resection for colorectal cancer.

The rapid shallow breathing index (RSBI) is widely used in the evaluation of ventilator withdrawal in the intensive care unit, although it is not fully applicable to early extubation following surgery and may provide falsely positive extubation criteria. [14] The diaphragm is the most important respiratory muscle, responsible for 60–70% of the body’s respiratory dynamics. Previously, diaphragmatic ultrasound was mostly used to predict the extubation outcome of patients in the intensive care unit, but the critical values would differ. In the intensive care unit, [15, 16] a DE-QB of 11–14 mm is considered to be the most sensitive and specific critical value in predicting the failure of extubation, whereas a DTF-DB of 36–40% is considered to be the most important value for predicting extubation success.

Extubating patients from anesthesia has been made easier with the advent of diaphragmatic ultrasonography. Lang confirmed that residual muscle relaxation can be determined by diaphragmatic ultrasound; [17] however, the research participants were non-thoraco-laparoscopic patients. The participants in the current study were elderly patients who had undergone laparoscopic colorectal cancer surgery. The Trendelenberg position and pneumoperitoneum pressure during the surgery damaged the diaphragm. [18, 19] Studies [20] have shown that even if extubation is performed under TOFr > 0.9, postoperative diaphragmatic ultrasound can still confirm the presence of residual muscle relaxation. There is still some risk involved if only a single indicator is used to instruct the extubation of elderly patients post-surgery.

In this study, we used diaphragmatic ultrasound parameters to predict the occurrence of CREs post-extubation in elderly patients after laparoscopic radical resection for colorectal cancer. We found that the DE-DB and DTF-DB could predict CREs after extubation in this set of patients. Muscle relaxation monitoring and individual diaphragmatic ultrasound parameters have been shown to predict prognosis; in this study, we combined these three, and our ROC curve analysis confirmed that the combined indicators had a predictive value for adverse events post-extubation in elderly patients, and the sensitivity and specificity of prediction were higher than those of any single indicator. This finding suggested that elderly patients undergoing laparoscopic radical resection for colorectal cancer might benefit from monitoring muscular relaxation in conjunction with diaphragmatic ultrasonography to help anticipate the incidence of CREs following extubation. The use of multiple indicators not only exerts the advantages of simple and rapid evaluation but also improves the efficiency of combined prediction. In this study, diaphragm ultrasound combined with muscle relaxation monitoring predicted that respiratory adverse events after extubation could be intervened in advance to reduce the discomfort of patients after extubation and promote their recovery. At the same time, high-risk patients with respiratory adverse events after extubation should be extubated more carefully. In the future, we can explore whether it can be used to predict respiratory adverse events in high-risk patients after extubation, so as to reduce the pain of patients and improve their prognosis.

There are also several limitations to this study. First of all, we selected the right diaphragm for measuring the diaphragm muscle value in this study and did not compare it with the left diaphragm since the stomach may affect the measurement. Secondly, the accuracy of the muscle relaxation monitor can be compromised by the discomfort it causes in patients who are conscious. Finally, this is a single-center, small-sample study; a large-sample prospective study is required to determine the utility of a combination of diaphragmatic ultrasound and muscle relaxation monitoring for predicting adverse respiratory events post-extubation in elderly patients.

## Conclusion

In conclusion, low muscle relaxation value, decreased diaphragm mobility during extubation and decreased diaphragm thickening rate during extubation can lead to adverse respiratory events in elderly patients after laparoscopic radical resection of colorectal cancer. We identified muscle relaxation monitoring, DE-DB, and DTF-DB during extubation as factors influencing adverse respiratory events post-extubation in elderly patients following laparoscopic radical resection for colorectal cancer. The combination of these three indicators for the prediction of adverse respiratory events after extubation showed higher sensitivity and specificity than single-indicator prediction, and this can offer new ideas and new methods for future clinical use in this population.

## Data Availability

The datasets used and/or analysed during the current study available from the corresponding author on reasonable request.
